# Immediate breast reconstruction surgery for breast cancer: current status and future directions

**DOI:** 10.1007/s12282-025-01723-5

**Published:** 2025-05-26

**Authors:** Tadahiko Shien, Hiroko Nogi, Akiko Ogiya, Makoto Ishitobi, Chikako Yamauchi, Ayaka Shimo, Kazutaka Narui, Naomi Nagura, Hirohito Seki, Kaori Terata, Miho Saiga, Tatsuya Uchida, Shinsuke Sasada, Teruhisa Sakurai, Naoki Niikura, Hiroki Mori, the Japanese Breast Cancer Society’s Collaborative Study Group of Scientific Research

**Affiliations:** 1 Department of Breast and Endocrine Surgery, Okayama University Hospital, 2-5-1 Shikata-Cho, Kita-Ku, Okayama, 7008558 Japan; 2 Department of Breast and Endocrine Surgery, The Jikei University School of Medicine, Tokyo, Japan; 3 Department of Breast Surgery, Japanese Red Cross Medical Center, Tokyo, Japan; 4 Department of Breast Surgical Oncology, Cancer Institute Hospital, Japanese Foundation for Cancer Research, Tokyo, Japan; 5 Department of Breast Surgery, Mie University School of Medicine, Mie, Japan; 6 Department of Breast Surgery, Osaka Habikino Medical Center, Osaka, Japan; 7 Department of Radiation Oncology, Shiga General Hospital, Shiga, Japan; 8 Department of Breast and Endocrine Surgery, St. Marianna University School of Medicine, Kanagawa, Japan; 9 Department of Breast and Thyroid Surgery, Medical Center, Yokohama City University, Kanagawa, Japan; 10 Department of Breast Surgical Oncology, St Luke’s International Hospital, Tokyo, Japan; 11 Department of Breast Surgery, Kyorin University School of Medicine, Tokyo, Japan; 12 Department of Breast and Endocrine Surgery, Akita University Hospital, Akita, Japan; 13 Department of Plastic Surgery, Okayama University Hospital, Okayama, Japan; 14 Department of Surgical Oncology, Research Institute for Radiation Biology and Medicine, Hiroshima University, Hiroshima, Japan; 15 Sakurai Breast Clinic, Wakayama, Japan; 16 Department of Breast Oncology, Tokai University School of Medicine, Kanagawa, Japan; 17 Department of Plastic and Reconstructive Surgery, Tokyo Medical and Dental University, Tokyo, Japan

**Keywords:** Breast cancer, Immediate reconstruction surgery, Prognosis, Complications

## Abstract

**Background:**

Immediate breast reconstruction (IBR) has become increasingly recognized in Japan as an important component of breast cancer care, improving patients’ quality of life after mastectomy. While the adoption of IBR is growing, the reconstruction rate in Japan remains lower than in Western countries. To clarify the current practice and challenges, the Japanese Breast Cancer Society (JBCS) conducted a nationwide survey.

**Methods:**

We conducted a comprehensive web-based questionnaire survey among all JBCS-certified institutions between December 2020 and February 2021. The survey assessed institutional capabilities, surgical techniques, decision-making criteria for BR, and the integration of adjuvant therapy.

**Results:**

A total of 429 institutions responded, with 72.5% offering BR and 61.7% capable of providing immediate reconstruction. Nipple-sparing mastectomy (NSM) was performed at 73.7% of institutions offering reconstruction. Multidisciplinary conferences with plastic surgeons were held at 70.5% of institutions. Approximately 30% of institutions discontinued IBR if sentinel lymph node metastases were detected intraoperatively, and 62.8% avoided recommending IBR for patients likely to require postoperative radiation therapy. In 94% of institutions, BR did not cause delays in the administration of adjuvant chemotherapy. However, 15% of institutions modified their radiation therapy approach in reconstructed patients. Additionally, 27% of physicians still believed that BR could negatively affect prognosis.

**Conclusions:**

The survey confirmed that IBR is widely performed and feasible in Japan. However, institutional differences, limited access to plastic surgeons, and persistent misconceptions remain significant barriers. Strengthening multidisciplinary collaboration and establishing standardized guidelines will help improve BR rates and patient outcomes in Japan.

**Supplementary Information:**

The online version contains supplementary material available at 10.1007/s12282-025-01723-5.

## Introduction

Breast reconstruction (BR) has become integral to breast cancer management worldwide, offering improved esthetic outcomes and enhancing patients’ Quality of Life (QOL). In Japan, the prevalence of BR has steadily increased, particularly following the national insurance coverage expansion for implant-based reconstruction in 2013 and prophylactic mastectomies in 2020 [1]. While the adoption of BR in Japan has been growing, the reconstruction rate remains significantly lower than in other countries [1, 2]. At the same time, only 18% of mastectomy patients in Japan underwent BR in 2018; the rate was considerably higher in South Korea at 53%. Factors contributing to this disparity include limited access to skilled plastic surgeons, concerns about oncological safety, and variability in multidisciplinary collaboration. Immediate breast reconstruction (IBR) has gained attention for its potential to restore the physical form immediately following mastectomy, minimizing the psychological impact of breast cancer surgery. With advances in surgical techniques, such as nipple-sparing mastectomy (NSM) and skin-sparing mastectomy (SSM), the esthetic outcomes have improved significantly. Despite these advancements, challenges remain, including concerns about surgical complications, integration with adjuvant therapies, and access to skilled plastic surgeons.

In Japan, unique cultural and systemic factors influence the adoption and implementation of BR. Cultural norms, such as a tendency to suppress sexual expression and discomfort with blunt, literal translations, may cause Japanese women to hesitate to answer questions about sexual health, which could lead to psychological resistance and lower response rates [3]. Limited availability of plastic surgeons, misconceptions about BR’s impact on prognosis, and logistical barriers to multidisciplinary collaboration pose considerable challenges. Furthermore, the rising use of neoadjuvant Chemotherapy (NAC) has introduced new considerations for the timing and safety of IBR.

## Background and literature context

Over the past several years, the Japanese Breast Cancer Society Scientific Research Group has conducted a series of investigations to better understand the role of BR in breast cancer management, particularly IBR, and its integration with adjuvant therapies. These efforts aimed to address specific challenges unique to the Japanese healthcare system and cultural landscape.

Subsequent multi-institutional research focused on the effects of radiation therapy in patients undergoing immediate BR. It was found that while PMRT slightly increased complication risks, careful planning and multidisciplinary collaboration could mitigate these effects. Approximately 15% of institutions reported modifying RT approaches to accommodate reconstruction, underscoring the complexities of balancing oncologic safety with reconstructive outcomes [2, 4].

Further studies assessed the prognosis of patients experiencing isolated locoregional recurrence after IBR. These results confirmed that recurrence did not significantly worsen long-term survival, aligning with international data supporting the oncologic safety of BR even after recurrence events [5]. The research group also explored complications specific to nipple-sparing mastectomy (NSM), such as nipple–areolar complex malposition. Multi-institutional studies emphasized the importance of surgical technique standardization and intraoperative assessment of the nipple margin to optimize both oncologic safety and esthetic outcomes.

Finally, the group reviewed evolving trends in breast cancer surgery, including increasing acceptance of breast-conserving surgery and the rising consideration of BR even after neoadjuvant chemotherapy. These analyses provided a comprehensive picture of changing patient preferences and the need for adaptive clinical strategies [6].

Through this body of work, the Japanese Breast Cancer Society Scientific Research Group has established a robust evidence base supporting the safety, feasibility, and patient-centered value of immediate BR in Japan. Building upon these findings, the present study aims to consolidate knowledge, address remaining barriers, and propose strategic directions for optimizing BR practices nationwide.

This aims to provide a comprehensive analysis of the current state of BR in Japan, focusing on surgical techniques, oncological outcomes, and patient satisfaction. Additionally, we will address the challenges unique to Japan, including physician and patient perspectives, and propose future directions for improving BR accessibility and outcomes. To identify the optimal Breast reconstruction surgery (BRS), the Japanese Breast Cancer Society conducted a group study of BRS in 2020, and this article seeks to guide clinicians and policymakers toward optimizing BR practices in Japan by synthesizing the latest evidence and expert consensus. This report serves as the final report summarizing the findings of this large-scale research project.

## Methods

This study was approved by the Japanese Breast Cancer Society (JBCS) scientific committee, and individual ethics committees belong to the authors (Okayama University #2101-028). A nationwide web-based questionnaire survey was conducted to assess BRS practices and the integration of adjuvant therapy in patients with operable breast cancer (Appendix 1). The survey was distributed to all 1010 JBCS-certified institutions between December 2020 and February 2021. Respondent institutions were those with JBCS-certified breast cancer specialists. This report primarily analyzes trends in BRS, institutional practices, decision-making criteria, and the impact on adjuvant therapy based on the survey responses.

## Results

### Breast reconstruction surgery (BRS)

A total of 429 institutions completed the questionnaire. The institutional breakdown included university hospitals (18.6%), public general hospitals (39.6%), private hospitals (26.1%), and private clinics (24.2%) (Table 1). BRS was performed at 72.5% (312/429) of these institutions, and prophylactic breast surgery was offered at 36.6%. BRS was most frequently performed at university hospitals (96.3%) and private hospitals (96.3%). Regional variation was observed, with the highest rates in the Kinki region (82.4%) and the lowest in the Hokuriku region (58.8%) (Table 2).

**Table 1:** Institutions that can be performed breast reconstruction surgery (*n* = 429)

	*n* (%)
University hospital	80 (18.6)
Public general hospital	170 (39.6)
Private general hospital	112 (26.1)
Private hospital/clinic	104 (24.2)

**Table 2:** Reconstruction surgery according to regions (based on the proportion of institutions that responded to the questionnaire, not individual cases)

Region	No. of responding institutions	Institutions performing reconstruction	Reconstruction rate (%)	Institutions performing NSM	NSM rate (%)
Hokkaido	23	16	69.6	11	68.6
Tohoku	31	24	77.4	14	58.3
Kanto-Koshinetsu	126	96	76.2	69	71.9
Tokai	60	36	60	22	61.1
Hokuriku	17	10	58.8	8	80
Kinki	85	70	82.4	64	91.4
Chugoku	28	20	71.4	19	95
Shikoku	15	9	60	5	55.6
Kyushu	44	32	72.7	18	56.2
Total	429	312	72.6	231	71.8

At institutions performing BRS, 80.1% had more than two breast surgeons, 87% had plastic surgeons, and 82.6% had radiation oncologists available. In addition, 59.3% (185/312) of these institutions performed more than 100 breast cancer surgeries annually, and 70.5% (220/312) reported conducting multidisciplinary conferences with plastic surgeons.

**Fig. 1:**
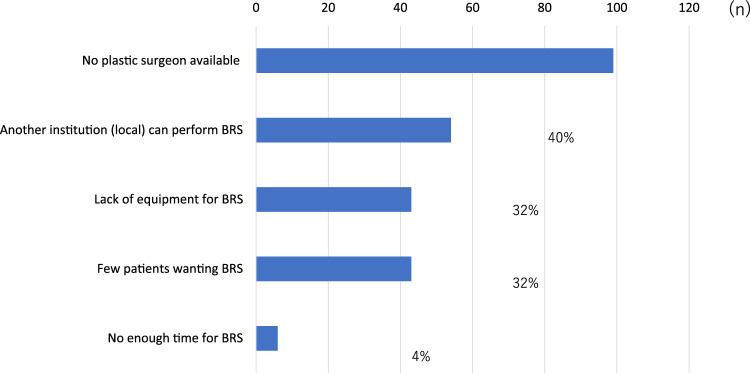
Reasons for not performing breast reconstruction after partial mastectomy

Immediate BRS was available in 61.7% (265/429) of institutions, and autologous reconstruction was possible at 52.9% (227/429). The types of reconstruction procedures offered are shown in Table 3. Reconstruction following partial mastectomy was less common (19.3%; 60/312), with the primary reasons for not offering BRS after breast-conserving surgery being the lack of plastic surgeons (73.3%), insufficient equipment (31.9%) and limited patient demand (31.9%) (Fig. 1).

**Table 3:** Types of breast reconstruction procedures available at participating institutions, including implant-based and autologous techniques

	Immediate and delayed surgery (*n* = 312) (%)	Immediate surgery (*n* = 301) (%)
TE/IMP	292 (92.4)	272 (88)
LD	227 (71.8)	198 (64.1)
DIEP	150 (47.5)	122 (39.5)
Fat implant	46 (14.6)	13 (4.2)

### Skin-sparing mastectomy procedures for BRS

**Fig. 2:**
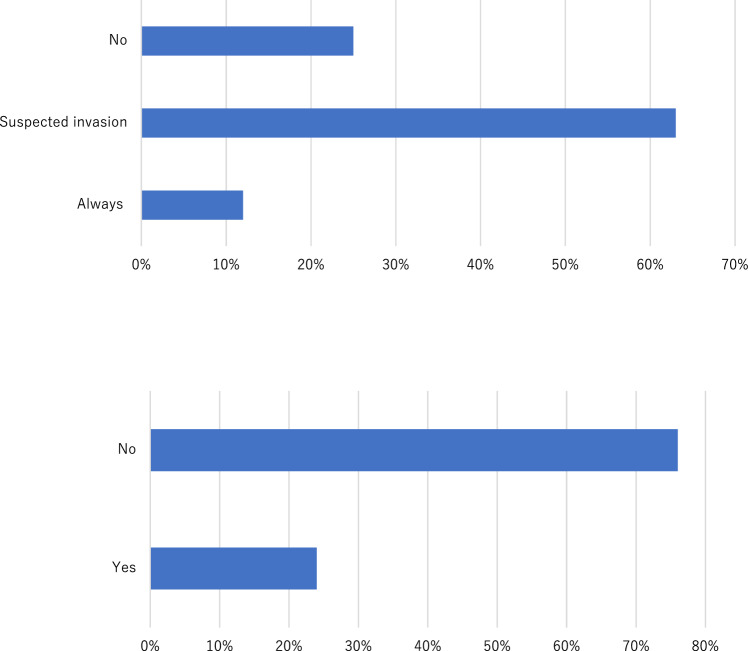
Institutional practices regarding nipple-sparing mastectomy (NSM). **A** Do you remove the skin over the tumor? **B** Do you remove the puncture point of needle biopsy?

Nipple-sparing mastectomy (NSM) was performed at 73.7% (230/312) of institutions offering BRS. Among these, 94% (216/230) had at least one plastic surgeon, and 70.9% (163/230) held multidisciplinary conferences with plastic surgeons. Intraoperative pathological assessment of the nipple margin was performed at 85.2% of institutions. Additionally, 62.8% (196/312) of institutions routinely excised skin-overlying tumors suspected of invasion. Skin at needle biopsy puncture sites was routinely removed by 24% (76/312) of institutions and selectively removed by 12% (36/312) (Fig. 2). Approximately half of the institutions reported preserving thicker skin flaps during reconstruction to optimize esthetic outcomes.

### Criteria for BRS and management of high-risk cases

**Fig. 3:**
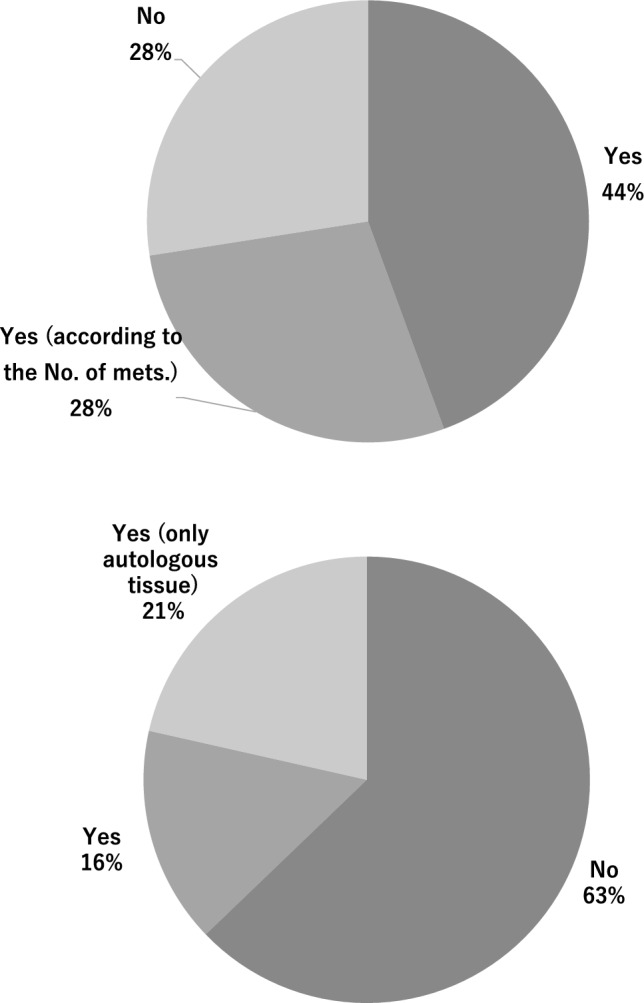
Institutional decision-making criteria regarding immediate breast reconstruction (IBR) in the presence of sentinel lymph node metastases and recommendations for patients requiring post-mastectomy radiation therapy (PMRT). **A** Will immediate reconstruction be performed even if metastasis is detected in the sentinel lymph nodes? **B** Will immediate reconstruction be performed even if postoperative radiation therapy is necessary?

Among BRS-performing institutions, 30.4% (95/312) reported discontinuing immediate BRS if sentinel lymph node metastasis was diagnosed intraoperatively (Fig. 3A), and 62.8% (196/312) did not recommend immediate BRS for patients with multiple lymph node metastases who would likely require postoperative RT (Fig. 3B).

When considering patients who had undergone NAC, 26% (85/312) of institutions performed BRS for those requesting reconstruction, whereas 31% (99/312) avoided it in this setting, no institutions offered BRS to patients with progressive disease after NAC, and 31% refrained from offering it to patients with stable disease.

### Impact on adjuvant therapy and RT planning

94% of institutions reported that BRS did not influence the administration of adjuvant chemotherapy. However, 15% of institutions reported modifying RT planning in reconstructed patients, often by reducing irradiation fields or omitting radiation in certain situations to avoid complications associated with irradiating reconstructed breasts, especially with implants. Data from additional survey results (*n* = 49) showed that approximately half of the institutions altered their RT approach post-BRS, and some institutions generally avoided RT following reconstruction.

61% (190/312) of institutions considered RT for patients with implant-based BRS, and 20% allowed RT to be administered to patients with tissue expanders in place. In 76.3% (238/312) of institutions, the criteria for postoperative RT were not changed based on the presence of reconstruction. Of these institutions, 63% (150/238) conducted multidisciplinary discussions with radiation oncologists.

### Postoperative follow-up

**Fig. 4:**
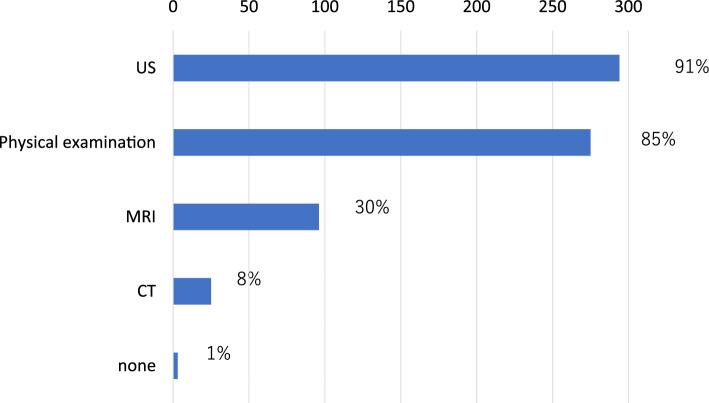
Routine postoperative follow-up practices after breast reconstruction

**Fig. 5:**
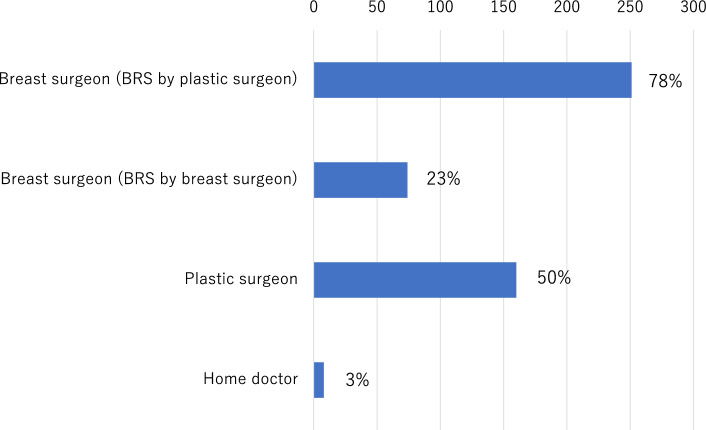
Distribution of responsibility for follow-up care after breast reconstruction

Figure 4 summarizes routine postoperative follow-up practices after BRS. While plastic surgeons provided follow-up care in 50% of institutions, breast surgeons remained primarily responsible for postoperative monitoring in most cases (78%) (Fig. 5).

### Physician perceptions

Approximately 27% of surveyed physicians believed that BRS negatively impacts patient prognosis despite evidence to the contrary. This perception was more common among female physicians and those practicing in low-volume breast cancer centers.

## Discussion

This nationwide survey conducted by the Japanese Breast Cancer Society (JBCS) scientific research group offers valuable and comprehensive insights into current clinical practices, physician perceptions, and challenges regarding BR and its integration with adjuvant therapy in Japan. According to the survey, 72.5% of responding institutions offered BR, with 61.7% capable of IBR and 52.9% performing autologous reconstructions [1]. Implant-based reconstruction remains the most common approach, especially among younger patients and those undergoing prophylactic mastectomy as it is less invasive and allows for faster recovery [1].

Despite the general availability of BR in Japan, several significant barriers impede its broader adoption. Shortages of plastic surgeons, lack of specialized equipment, and regional disparities in patient access continue to pose challenges [1]. Furthermore, 30.5% of institutions reported discontinuing IBR when sentinel lymph node metastases were detected intraoperatively, and 67% avoided recommending IBR for patients anticipated to require post-mastectomy radiation therapy (PMRT) [1]. These practices often reflect cautious decision-making influenced by institutional policies or individual physician preferences rather than robust evidence. Japanese clinical guidelines before 2018 also cautioned that post-reconstruction RT could potentially lead to complications or other adverse outcomes for patients. However, subsequent studies have shown that while PMRT may increase the rate of complications in reconstructed breasts, these risks can be effectively managed through appropriate planning and patient selection [2].

The survey also identified variability in RT planning. Notably, 15% of institutions adjusted RT delivery by reducing irradiation fields or omitting RT altogether in certain situations [1]. This likely reflects concerns over complications associated with irradiating reconstructed breasts, a pattern consistent with findings from international reports [7]. However, large-scale studies confirm that long-term outcomes and local recurrence (LR) rates after BR are comparable to those following breast-conserving surgery even when RT is administered [4].

An increasing trend toward nipple-sparing mastectomy (NSM) was observed, with 75.2% of BR-offering institutions performing NSM [1]. Although NSM improves esthetic outcomes, it also introduces technical challenges. Malposition of the nipple–areolar complex remains a frequent complication, emphasizing the need for standardized surgical protocols and advanced training [8]. Additionally, a detailed classification of local recurrence patterns post-NSM has revealed that nipple–areolar recurrences differ from other LR types, underscoring the importance of tailored surveillance strategies [8].

It is encouraging that most patients undergoing IBR commenced adjuvant therapy within three months post-surgery, meeting a clinically critical time window associated with improved prognosis [9]. Nonetheless, misconceptions remain prevalent, with approximately 27% of physicians believing that BR adversely affects patient outcomes, a perception more common among female physicians and those working in low-volume centers [1]. These findings highlight the ongoing need for educational initiatives and the dissemination of evidence-based guidelines to address misconceptions about BRS’s oncologic safety. Multi-institutional studies have demonstrated that isolated locoregional recurrence after IBR does not compromise long-term prognosis [9]. Additionally, data on patient-reported outcomes, including sexual well-being after BR in the Japanese population, emphasize the psychological and quality-of-life benefits of reconstruction [3].

The overall BR rate in Japan continues to lag behind Western countries, where rates exceed 40–50% [1]. Cultural factors—such as a strong emphasis on complete cancer eradication and hesitancy toward implants—likely contribute to this disparity [10]. Many patients still choose total mastectomy over breast-conserving surgery, even when eligible, and may refuse additional surgery following complete clinical response to neoadjuvant chemotherapy [5]. Historical registry data also reveal gradual but considerable changes in breast cancer management trends over time [11]. Importantly, patient preferences for surgical approaches in cases of recurrence have been increasingly studied, suggesting evolving attitudes toward surgery and BR [12]. Furthermore, recent reviews of adjuvant and neoadjuvant therapy have outlined new developments and their impact on surgical choices and reconstruction planning [13].

## Conclusion

This nationwide survey revealed that BR is widely available in Japan, with most institutions capable of providing immediate and autologous rebuilding. Institutional variations, surgeon-dependent decision-making, and persistent misconceptions about the oncological safety of BR and its relationship with radiation therapy remain obstacles. Additionally, Japan’s relatively low BR rate compared to Western countries appears to be influenced by systemic limitations and cultural attitudes. Standardized clinical guidelines, robust multidisciplinary collaboration, and comprehensive education for healthcare professionals and patients are essential to address these challenges. Continued research and nationwide efforts will be necessary to ensure safe, equitable, and patient-centered access to BR in Japan.

## Supplementary Information

Below is the link to the electronic supplementary material.

Supplementary file1 (DOCX 30 KB)
