# Supplementary material for: Immediate breast reconstruction surgery for breast cancer: current status and future directions
Source: Breast Cancer. 2025 May 26;32(4):630–7. doi: 10.1007/s12282-025-01723-5 (PMC12174185; doi:10.1007/s12282-025-01723-5)
Supplement: Supplementary file 1 — Supplementary file1 (DOCX 30 KB) [file 12282_2025_1723_MOESM1_ESM.docx]

# Appendix1 Structure of the Breast Reconstruction Survey Questionnaire

| Section | Survey Items |
| --- | --- |
| Institution Information | - Number of breast surgeons - Number of plastic surgeons - Number of radiation oncologists - Multidisciplinary conference between breast and plastic surgeons - Multidisciplinary conference between breast surgeons and radiation oncologists |
| Breast Reconstruction (BRS) | - Whether BRS is performed - Timing of reconstruction (immediate, delayed, both) - Types of reconstruction (implant-based, autologous, others) - Methods used for immediate (single-stage or two-stage) reconstruction - Number of breast surgeries in 2019 - Number of mastectomies - Number of immediate reconstructions - Number of delayed reconstructions |
| Patient Selection Criteria | - Immediate reconstruction for patients with lymph node metastases - Immediate reconstruction in patients expected to require postoperative radiation therapy (PMRT) - Immediate reconstruction after neoadjuvant chemotherapy (NAC) - Eligibility criteria for reconstruction after NAC:  * Pre-NAC tumor (T) factor  * Pre-NAC nodal (N) factor  * Pre-NAC stage  * Response to NAC - Breast reconstruction after partial mastectomy - Reconstruction methods for partial mastectomy |
| Nipple-Sparing Mastectomy (NSM) | - Whether NSM is performed - Selection criteria for NSM (multiple selections allowed) - Intraoperative frozen section pathology for the nipple base |
| Surgical Techniques | - Management of skin overlying tumors - Management of skin at needle biopsy or vacuum-assisted biopsy sites - Type of flaps used in immediate reconstruction |
| Radiation Therapy | - Indications for PMRT in cases with:  * 1–3 positive lymph nodes  * 4 or more positive lymph nodes  * Tumors larger than 5 cm - Inclusion of internal mammary nodes in radiation fields - PMRT after NAC - Changes in RT planning after reconstruction:  * Tissue expander in place  * Implant in place  * After autologous reconstruction  * RT to the nipple-areolar complex in NSM cases - Complications observed in PMRT after reconstruction |
| Postoperative Follow-up | - Regular imaging and examination methods for reconstructed breasts |
| Additional Information | - Frequency of surgical site infections after immediate reconstruction following NAC |
